# Immunohistochemical and transcriptome analyses indicate complex breakdown of axonal transport mechanisms in canine distemper leukoencephalitis

**DOI:** 10.1002/brb3.472

**Published:** 2016-05-03

**Authors:** Ingo Spitzbarth, Charlotte Lempp, Kristel Kegler, Reiner Ulrich, Arno Kalkuhl, Ulrich Deschl, Wolfgang Baumgärtner, Frauke Seehusen

**Affiliations:** ^1^ Department of PathologyUniversity of Veterinary Medicine Hannover FoundationBünteweg 1730559HannoverGermany; ^2^ Center for Systems NeuroscienceBünteweg 230559HannoverGermany; ^3^ Department of Non‐Clinical Drug SafetyBoehringer Ingelheim Pharma GmbH & Co KGBiberach (Riß)Germany

**Keywords:** Growth‐associated protein 43, microarray, neurofilament, p75 neurotrophin receptor, periaxin, Schwann cell

## Abstract

**Introduction:**

CDV‐DL (Canine distemper virus‐induced demyelinating leukoencephalitis) represents a spontaneously occurring animal model for demyelinating disorders. Axonopathy represents a key pathomechanism in this disease; however, its underlying pathogenesis has not been addressed in detail so far. This study aimed at the characterization of axonal cytoskeletal, transport, and potential regenerative changes with a parallel focus upon Schwann cell remyelination.

**Methods:**

Immunohistochemistry of canine cerebellar tissue as well as a comparative analysis of genes from an independent microarray study were performed.

**Results:**

Increased axonal immunoreactivity for nonphosphorylated neurofilament was followed by loss of cytoskeletal and motor proteins. Interestingly, a subset of genes encoding for neurofilament subunits and motor proteins was up‐regulated in the chronic stage compared to dogs with subacute CDV‐DL. However, immunohistochemically, hints for axonal regeneration were restricted to up‐regulated axonal positivity of hypoxia‐inducible factor 1 alpha, while growth‐associated protein 43, erythropoietin and its receptor were not or even down‐regulated. Periaxin‐positive structures, indicative of Schwann cell remyelination, were only detected within few advanced lesions.

**Conclusions:**

The present findings demonstrate a complex sequence of axonal cytoskeletal breakdown mechanisms. Moreover, though sparse, this is the first report of Schwann cell remyelination in CDV‐DL. Facilitation of these very limited endogenous regenerative responses represents an important topic for future research.

## Introduction

CDV‐DL (Canine distemper virus‐induced demyelinating leukoencephalitis) represents the most frequent manifestation of the central nervous form of distemper in dogs (Beineke et al. 2009). Though primarily virus induced, due to morphological findings, CDV‐DL has been suggested as a spontaneous animal model for human demyelinating diseases such as MS (multiple sclerosis) (Seehusen and Baumgärtner 2010; Spitzbarth et al. 2012).

Demyelination during CDV‐DL appears to represent a biphasic process with a primary virus‐induced oligodendroglial dystrophy followed by a secondary wave of immune‐mediated myelin destruction (Vandevelde et al. 1982; Ulrich et al. 2014). Substantiating this hypothesis, a recent microarray study of CDV‐DL in canine brain tissue identified numerous up‐regulated genes participating in processes of the innate and the humoral immune response (Ulrich et al. 2014). In addition, multiple myelin genes including MBP (myelin basic protein) and proteolipid protein displayed a selective down‐regulation in subacute CDV‐DL, suggestive of oligodendrocyte dystrophy (Ulrich et al. 2014). In contrast, multiple genes involved in the adaptive immune response were up‐regulated in chronic inflammatory lesions of CDV‐DL, thus favoring the hypothesis of bystander immune‐mediated demyelination in the advanced phase of the disease.

Similar to MS, CDV‐DL has long been regarded as a primary demyelinating and inflammatory disease. However, more recently, axonal pathology, characterized by accumulation of nNF (nonphosphorylated neurofilament) and beta APP (amyloid precursor protein), has been demonstrated to represent a hallmark of CDV‐DL (Seehusen and Baumgärtner 2010; Imbschweiler et al. 2012). Interestingly, axonal damage precedes myelin loss in CDV‐DL, thus indicating that axonopathy represents an early key pathomechanism, which potentially functions as an initial triggering factor for subsequent events such as demyelination and inflammation (Seehusen and Baumgärtner 2010; Imbschweiler et al. 2012; Lempp et al. 2014). This observation is in line with the so called “inside‐out theory” of demyelination, that is, primary axonal damage with subsequent secondary loss of myelin sheaths (Tsunoda and Fujinami 2002; Tsunoda et al. 2003).

Despite recent progress in understanding axonal damage as a so far underestimated pivotal event in demyelinating diseases, knowledge about the underlying molecular disturbances, contributing to axonopathy is sparse. Proper axonal function depends on the integrity of various cytoskeletal constituents, motor‐, and microtubule‐associated proteins. In fact, NF (neurofilament) expression is modulated at multiple levels. Synthesized in the perikaryon, NFs undergo local phosphorylation along with axonal transport. Axonal cargo is carried out along microtubules and their anterograde and retrograde movement is dependent on association with the motor proteins kinesin and dynein, respectively. In particular, kinesin family member 5A (KIF5A) is a neuron‐specific protein, responsible for the transport of axonal components such as NF, APP, and cell organelles such as mitochondria (Hares et al. 2013). Interestingly, in MS and TMEV‐IDD (Theiler's murine encephalomyelitis virus‐induced demyelinating disease), there is evidence of decreasing amounts of KIF5A, indicating impaired axonal transport processes in these diseases (Kreutzer et al. 2012; Hares et al. 2013). The MAPT (microtubule‐associated protein tau) represents a key protein, which conciliates the interactions of microtubules and motor proteins, respectively. Consequently, a reduction in MAPT expression could facilitate a hypophosphorylated NF microtubule‐dependent axonal transport, as the association of hypophosphorylated NF with microtubules is critically dependent on the amount of MAPT (Shah et al. 2000).

In contrast to the historical dogma of irreversibility of damage to CNS (central nervous system) axons, there is increasing evidence of endogenous regenerative events following various neurological disorders such as traumatic CNS injury (Schwab and Bartholdi 1996). For instance, axons in MS plaques as well as in SCI (spinal cord injury) in humans and dogs express GAP43 (growth‐associated protein 43), a protein which is involved in axonal development, regeneration, and outgrowth (Li et al. 1996; Schwab and Bartholdi 1996; Bock et al. 2013; Schirmer et al. 2013). The regenerative outgrowth of mature axons resembles the growth of a developmental growth cone in many aspects and is associated with cytoskeleton remodeling, microtubule dynamics, and NF plasticity (Erez and Spira 2008; Bradke et al. 2012; Gordon‐Weeks and Fournier 2014). Furthermore, certain factors such as EPO (erythropoietin) and its receptor (EPOR) have been shown to act as neuroprotective factors in the CNS in various CNS pathologies including demyelinating disease (Ehrenreich et al. 2007; Hagemeyer et al. 2012). Hypoxia‐inducible factor‐1 alpha (HIF1A) accumulates in the CNS for instance under conditions of hypoxia in the rodent model (Wiener et al. 1996; Bergeron et al. 1999; Pascual et al. 2001). This transcription factor pivotally influences multiple target genes including EPO and thus its up‐regulation might exert neuroprotective effects (Xiaowei et al. 2006).

Similar to axonal regeneration, there are attempts to restore myelin in demyelinating diseases such as MS. In fact, remyelination has been described in MS and some of its animal models such as TMEV‐IDD (Patrikios et al. 2006; Franklin and Ffrench‐Constant 2008; Crawford et al. 2013; Raddatz et al. 2016). Despite such attempts, remyelination by oligodendrocytes is believed to fail due to the inhibition of differentiation from precursors to mature oligodendrocytes (Levine and Reynolds 1999; Franklin and Ffrench‐Constant 2008; Ulrich et al. 2008; Sun et al. 2015). Besides oligodendrocytes, Schwann cells represent another putative cell type, which has been shown to remyelinate demyelinated areas in CNS diseases such as MS and its experimental models as well as SCI (Ghatak et al. 1973; Dal Canto and Lipton 1980; Itoyama et al. 1983; Blakemore 2005; Powers et al. 2013). In CDV‐DL, axonopathy parallels an early emergence of p75^NTR^‐positive bipolar glial cells (Imbschweiler et al. 2012). Interestingly, a specific population of growth‐promoting macroglia, referred to as aldynoglia, shares morphological and molecular properties with peripheral premyelinating Schwann cells and similarly expresses p75^NTR^ (Gudino‐Cabrera and Nieto‐Sampedro 2000; Orlando et al. 2008; Spitzbarth et al. 2015). Thus, it was proposed that the cells detected in CDV‐DL lesions might possibly represent a premyelinating stage of Schwann cells (Imbschweiler et al. 2012). However, evidence of effective Schwann cell remyelination has not been shown so far in CDV‐DL.

This study aimed to precisely characterize the temporal development of axonal injury during CDV‐DL with a special emphasis upon the axoskeleton and axonal transport involvement. Moreover, we sought to determine hints for possible spontaneous regenerative events including expression of axonal regeneration‐promoting factors as well as evidence for Schwann cell‐mediated remyelination. The presented data provide a basis for future studies on regenerative and degenerative pathomechanisms during demyelinating diseases and may help to identify possible therapeutic targets in spontaneous demyelinating diseases.

## Materials and Methods

### Morphological examination

This study was conducted in accordance with the German Animal Welfare Act. No animals were infected or killed for this retrospective pathological case–control study. No animal experiments were performed since all investigations were performed on postmortem tissue, collected during routine necropsies.

**Figure 1:**
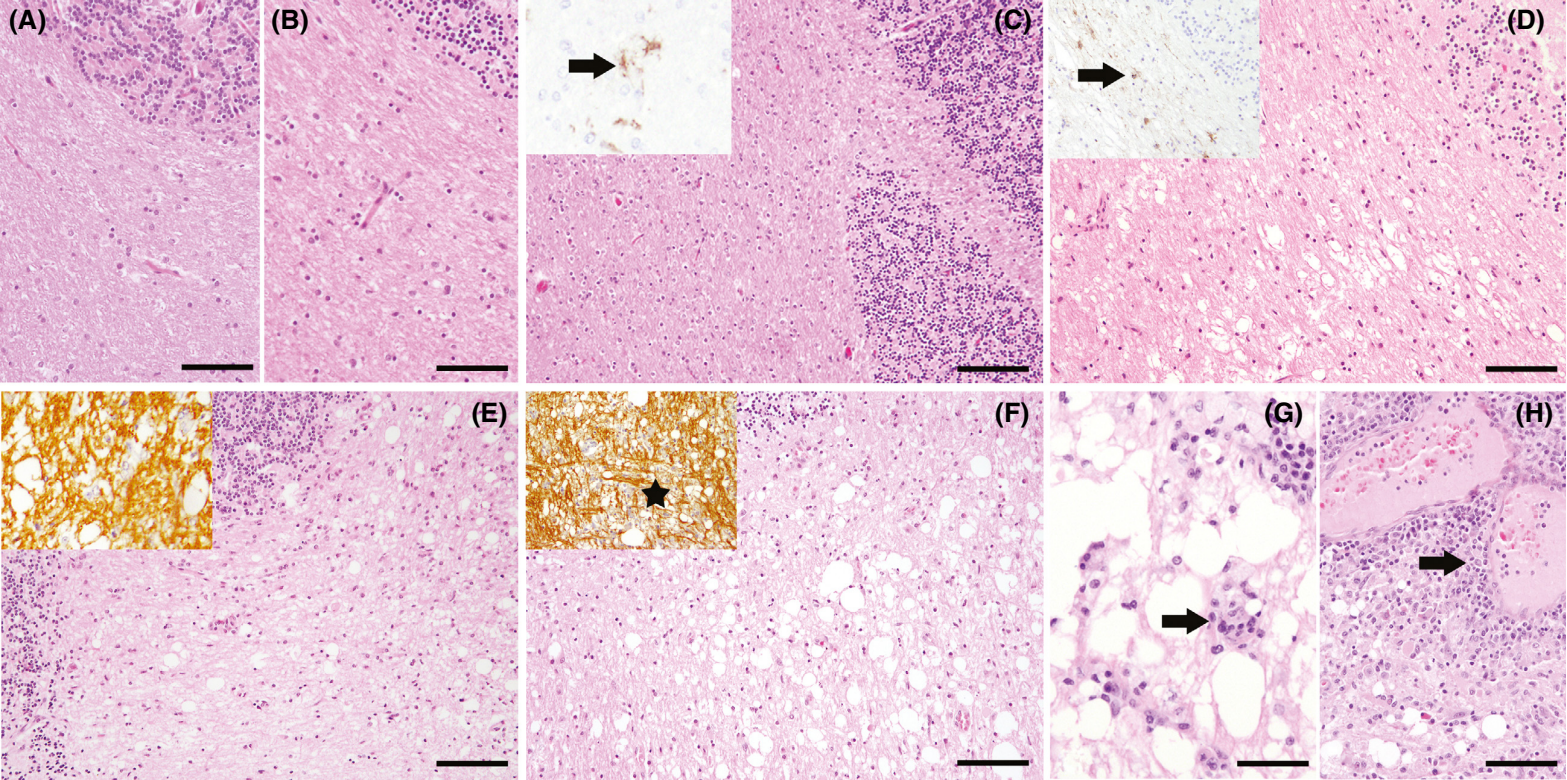
Histopathological classification of different distemper lesions. (A) Control animal without histopathological changes. HE stain, bar = 100 *μ*m. (B) Normal appearing white matter (NAWM) of a CDV‐infected animal without histopathological lesions. HE stain, bar = 100 *μ*m. (C) Cerebellar area without histopathological changes (antigen without lesion). HE stain, bar = 150 *μ*m. Inset shows immunohistochemical detection of CDV antigen in this white matter area (arrow). (D) Vacuolation, consisting of areas with myelin edema without inflammatory cell infiltration and gliosis. HE stain, bar = 100 *μ*m. Inset shows detection of CDV antigen‐positive cells by immunohistochemistry (arrow). (E) Acute lesion, characterized by focal vacuolation of the white matter without demyelination and absence of inflammation, but with mild to moderate gliosis. HE stain, bar = 100 *μ*m. Inset shows preservation of myelin. Immunohistochemistry for myelin basic protein (MBP). (F) Subacute lesion without inflammation displaying a mild to moderate pallor interpreted as decrease in myelin content and an increased number of reactive astrocytes, macrophages/microglial cells. HE stain, bar = 100 *μ*m. Inset shows loss of myelin (asterisk). Immunohistochemistry for MBP. (G) Subacute inflammatory lesion characterized by a loss of myelin, a mild perivascular infiltration with lymphohistiocytic cells (arrow) and scattered lymphocytes and macrophages throughout the lesion. HE stain, bar = 50 *μ*m. (H) Chronic lesion with prominent perivascular lymphohistiocytic infiltration of more than three layers of thickness (arrow) and marked demyelination with gitter cells. HE stain, bar = 100 *μ*m. Immunohistochemistry performed with avidin‐biotin‐peroxidase complex method with 3,3′‐diaminobenzidine as chromogen.

Archived cerebellar tissue of eight neurologically healthy control dogs and 17 male and female dogs of different breeds with spontaneous CDV‐DL was examined. The age of the study subjects ranged from 2 months to 7 years. The tissue from dogs with distemper originated from the routine necropsy material of the Departments of Pathology, University of Veterinary Medicine Foundation, Hannover, and the Justus‐Liebig‐University, Gießen, Germany. The tissue from three animals was kindly provided by Dr. Juan Alberto Morales, Servicio de Pathologia, Escuela de Medicina Veterinaria Universidad Nacional, Heredia, Costa Rica. All animals died spontaneously or were killed due to a poor prognosis. Tissues from four animals have been used in previously published studies (Seehusen and Baumgärtner 2010; Ulrich et al. 2014). Tissues were fixed in 10% non‐buffered formalin and embedded in paraffin wax, followed by preparation of 3‐*μ*m thick serial sections. Lesions were classified according to a morphology‐based classification scheme, which is widely used for CDV‐DL lesions (Vandevelde et al. 1982; Seehusen and Baumgärtner 2010; Imbschweiler et al. 2012). Based on morphological changes, the immunohistochemical demonstration of CDV antigen, and demyelination using an antibody against MBP, respectively, we classified seven distinct lesion groups (groups 2–8; *n* = 121; Fig. 1), while cerebellar white matter of nondiseased control animals was defined as group 1 (*n* = 24; Fig. 1A).

In the 17 diseased dogs, various lesion types occurred in parallel in each animal. Cerebellar lesions of group 2 (*n* = 13) were defined as cerebellar white matter areas of infected dogs, displaying no morphological changes in HE sections and no immunohistochemical evidence of CDV antigen (normal appearing white matter; NAWM; Fig. 1B). Group 3 lesions (*n* = 23) were defined as areas with CDV‐positive cells but no obvious lesion in the HE‐staining (antigen without lesion; Fig. 1C). Lesions, consisting of areas with vacuolation of the white matter caused by myelin edema but without inflammatory cell infiltration were defined as group 4 (*n* = 15; Fig. 1D). Group 5 consisted of acute lesions (*n* = 17), exhibiting mild vacuolation with mild astrogliosis and few microglia/macrophages (Fig. 1E). Subacute lesions without inflammation (group 6; *n* = 17) were defined as tissue areas with a decreased myelin density, malacic foci with gitter cells and activated microglia/macrophages (Fig. 1F). In subacute lesions with inflammation (group 7; *n* = 22), marked vacuolation and demyelination, infiltration with gitter cells and microglia/macrophages as well as perivascularly accentuated mononuclear infiltrates forming up to two perivascular layers were evident (Fig. 1G). Group 8 consisted of chronic lesions (*n* = 14) with severe demyelination, microglia/macrophages, and perivascular lymphohistiocytic cuffs of more than three cell layers (Fig. 1H). Eosinophilic intranuclear inclusion bodies, characteristic for CDV‐DL, were occasionally observed in groups 5–8.

### Immunohistochemistry and double immunofluorescence

For the immunohistochemical evaluation of viral antigen expression, axonal expression of various cytoskeletal, motor‐ and regeneration‐promoting proteins, and the identification of nonmyelinating and myelinating Schwann cells, respectively, the ABC (avidin‐biotin‐peroxidase‐complex; Vector Laboratories, Burlingame CA) method was performed as previously described (Seehusen et al. 2007; Bock et al. 2013), using a panel of mono‐ and polyclonal antibodies (Table 1). Demyelination was evaluated using an antibody against MBP. Antibodies detecting axonal cytoskeletal constituents comprised anti‐phosphorylated and ‐nonphosphorylated neurofilaments (pNF/nNF), anti‐acetylated alpha‐tubulin and ‐beta‐tubulin III. The expression of axonal motor proteins was evaluated using antibodies directed against kinesin (KIF5A) and dynein (intermediate chain of cytoplasmic dynein) as well as an antibody against the MAPT. Disturbed fast axonal transport processes were detected using an antibody against APP. For evaluation of axonal growth‐promoting factors, antibodies directed against growth‐associated protein 43 (GAP43), EPO and its receptor (EPOR) as well as an antibody against hypoxia‐inducible factor 1 alpha (HIF1A) were applied. Nonmyelinating Schwann cells were investigated using an antibody against p75 neurotrophin receptor (p75^NTR^) as described (Imbschweiler et al. 2012; Kegler et al. 2015). Lesions with evident infiltration of p75^NTR^‐positive bi‐ to multipolar cells were additionally investigated for the expression of PRX (periaxin), a myelin protein, which is expressed by myelinating Schwann cells (Kegler et al. 2015).

**Table 1:** Immunohistochemistry: antigens, clonality, and origin of the antibodies, dilution, and pretreatment

Detected antigen	Clonality, origin	Pretreatment	Dilution
CDV	mAB mouse, D110^a^	Microwave/CB 20 min	1:2000
MBP	pAB rabbit, Chemicon AB980	None	1:800
pNF	mAB mouse, Sternberger monoclonals SMI‐312R	None	1:4000
nNF	mAB mouse, Sternberger monoclonals SMI‐311R	Microwave/CB 20 min	1:1000
APP	mAB mouse, Chemicon MAB348	Microwave/CB 20 min	1:800
Beta‐tubulin III	mAB mouse, Sigma‐Aldrich T8660	Microwave/CB 20 min	1:1000
Acetylated alpha‐tubulin	mAB mouse, Sigma‐Aldrich T6793	None	1:500
Cytoplasmic Dynein Intermediate Chain	mAB mouse, Covance MMS‐400R	Microwave/CB 20 min	1:100
Kinesin 5A	pAB rabbit, Sigma‐Aldrich K0889	Microwave/CB 20 min	1:100
MAPT	mAB mouse, Millipore IHCR1015‐6	Microwave/CB 20 min	1:1000
GAP‐43	pAB rabbit, Millipore AB5220	Microwave/CB 20 min	1:600
EPO	pAB rabbit, R&D Systems AB‐286‐NA	Microwave/CB 20 min	1:200
EPOR	pAB rabbit, Santa Cruz sc‐695	Microwave/CB 20 min	1:100
HIF1A	pAB rabbit, Novus Biologicals NB100‐134	Microwave/CB 20 min	1:500
P75^NTR^	mAB mouse, ATCC HB8737	Microwave/CB 20 min	1:5
PRX	pAB rabbit, Sigma‐Aldrich HPA001868	Microwave/CB 20 min	1:5000

APP, beta‐amyloid precursor protein; CB, citrate buffer; CDV, canine distemper virus; EPO, erythropoietin; EPOR, erythropoietin receptor; HIF1A, hypoxia‐inducible factor 1*α*; mAB, monoclonal antibody; MBP, myelin basic protein; nNF, nonphosphorylated neurofilament; pAB, polyclonal antibody; pNF, phosphorylated neurofilament.

a Kindly provided by Prof. A. Zurbriggen; Vetsuisse faculty, University of Bern; Switzerland.

**Table 2:**
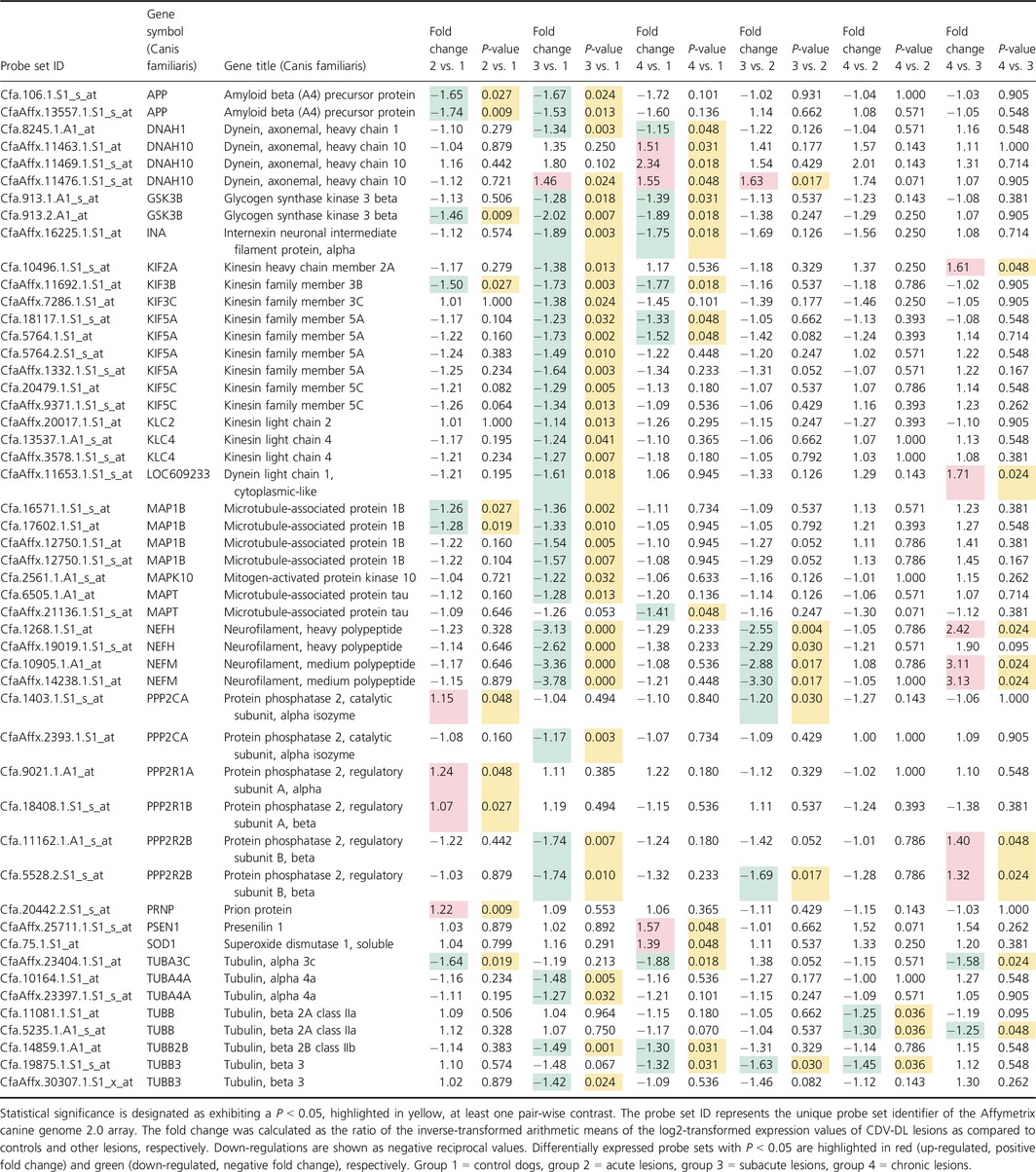
List of axon‐related differentially expressed probe sets, extracted from a previously published microarray data set (Ulrich et al. 2014)

Briefly, slides were dewaxed and hydrated with a graded series of alcohols. Pretreatment for antigen demasking was performed if necessary (Table 1). Endogenous peroxidase activity was blocked using 0.5% H_2_O_2_ in methanol. Subsequently, the sections were incubated overnight at 4°C with the respective primary antibody (Table 1). For negative controls, monoclonal antibodies were replaced by ascites fluid from nonimmunized Balb/C mice (dilution 1:1000) and polyclonal antibodies were substituted by serum from nonimmunized rabbits (dilution 1:3000). After incubation, the slides were treated with secondary antibodies (biotinylated goat anti‐mouse and biotinylated goat anti‐rabbit, respectively). Specific antigen–antibody binding was visualized using 3,3’‐diaminobenzidine‐tetrahydrochrloride (DAB) with 0.03% H_2_O_2_. Mayer's hematoxylin was used for counterstaining.

Immunopositivity was evaluated quantitatively. The expression of MBP, pNF, acetylated alpha‐tubulin, beta‐tubulin III, dynein, KIF5A, and MAPT was analyzed by morphometrical assessment of the immunopositive area (given in percentage of total area) using a Olympus BX‐51 digital camera microscope (Olympus Optical Co. (Europe) GmbH, Hamburg, Germany) and the software Analysis 3.1 (Soft Imaging System) (Bock et al. 2013). Expression of CDV antigen, APP, nNF, EPO, EPOR, HIF1A, GAP43, p75^NTR^, and PRX was quantitatively evaluated by counting the number of immunopositive axons and cells, respectively, in the entire lesion area using a morphometric grid (given as positive structures per square mm).

Representative sections with infiltration of p75^NTR^‐positive cells underwent double immunofluorescence according to previously published methodology (Kegler et al. 2015). Sections were analyzed for colocalization of p75^NTR^ with the transcription factor sex‐determining region Y‐box 2 (SOX2; 1:20, Cell Signaling Technology, Inc., Danvers, MA), glial fibrillary acidic protein (GFAP; 1:400, DakoCytomation, Hamburg, Germany), and PDGFR (platelet‐derived growth factor receptor)‐alpha (1:200, Santa Cruz Biotechnology, Inc., Dallas, TX). Briefly, sections were simultaneously incubated with the respective primary antibodies for 90 min. Cy3‐labeled goat anti‐mouse (red, 1:200, Alexa Fluor 555 dye, Life Technologies) and Cy2‐labeled goat anti‐rabbit (green, 1:200, Alexa Fluor 488 dye, Thermo Fisher Scientific GmbH, Dreieich, Germany) secondary antibodies were applied. Nuclear counterstaining was performed with 0.01% bisbenzimide (H33258, Sigma Aldrich, Taufkirchen, Germany) and sections were mounted with Dako fluorescent mounting medium (DakoCytomation, Hamburg, Germany). Moreover, lesions with PRX‐positive cells were representatively double labeled with an antibody against myelin protein 0 (clone P07, 1:400) (Archelos et al. 1993; Imbschweiler et al. 2012) using immunfluorescence.

### Transcriptome analysis of genes involved in axonal processes

In parallel, a literature‐based list of 79 genes (Table S2) referring to axonal cytoskeleton and transport processes as well as axonal regeneration was manually created (Kreutzer et al. 2012; Paus et al. 2006). Previously published murine and human genes, implied in axonal processes, were converted into orthologous canine gene symbols using the MADGene web tool (Baron et al. 2011); http://cardioserve.nantes.inserm.fr/madtools/madgene/. Furthermore, selected orthologous canine genes were retrieved using Information Hyperlinked over Proteins (Hoffmann and Valencia 2004); (http://www.ihop-net.org/UniPub/iHOP/). The respective data were extracted from a previously published and publically available Minimum Information About a Microarray Experiment (MIAME)‐compliant microarray data set upon CDV‐DL, which is based on GeneChip canine genome 2.0 arrays (Affymetrix, Santa Clara, CA) (Ulrich et al. 2014) (accession number: E‐MEXP‐3917; http://www.ebi.ac.uk/arrayexpress). This study was performed using RNA isolated from frozen brain sections control animals (control, *n* = 12) and 14 CDV‐infected dogs suffering from spontaneously occurring and immunohistologically confirmed CDV‐DL (Ulrich et al. 2014). The latter were classified as acute CDV leukoencephalitis (acute, *n* = 5), subacute CDV leukoencephalitis with demyelination but without inflammation (subacute, *n* = 6), and chronic CDV leukoencephalitis with demyelination and inflammation (chronic, *n* = 3) and all of these animals displayed only one lesion type in the processed brain areas (Ulrich et al. 2014). In this study, the normalized data set has been used, whose global analysis has previously been published and explained in full detail (Ulrich et al. 2010). In the original study, background adjustment, quantile normalization, and probe set summarization were performed using the GC‐RMA algorithm (Bioconductor gcrma for R package, version 2.3, www.bioconductor.org). Fold changes in this data set are given as the ratio of the inverse‐transformed arithmetic means of the log2‐transformed expression values between the respective groups (Ulrich et al. 2010). The publically available normalized global data set was filtered for the 79 literature‐based genes. This resulted in a list of 57 genes, represented on the GeneChip canine genome 2.0 array. The 57 unique canine gene symbols were represented by 135 probe sets on the array as some gene symbols were annotated by multiple probe sets on the array.

### Statistical analysis

Statistical analysis of the data from the immunohistochemical and transcriptome investigations was performed using SPSS (Superior Performing Systems, Version 22.0, IBM, New York, NY). As parts of the data were not normally distributed, nonparametric tests were applied. For immunohistochemical data, Kruskal–Wallis test was used to detect significant differences in antigen expression, followed by subsequent pair‐wise post hoc tests with alpha correction for multiple testing. In order to reveal potential codependencies in the expression of the antigens, correlation analysis was performed by calculating the Spearman's rank correlation coefficient. Only correlations coefficients of *r* > 0.7 and *r* < −0.7, respectively, and exhibiting a *P*‐value <0.05 were considered biologically relevant. The transcriptome data of manually extracted genes of interest were analyzed by nonstringent multiple pair‐wise Mann–Whitney *U*‐tests between the four respective groups (Ulrich et al. 2014).

Statistical significance was designated as *P* ≤ 0.05 in both the immunohistochemical and transcriptome investigations. Boxplots were created using GraphPad Prism version 5.0 for Windows (GraphPad Software Inc., La Jolla, CA).

## Results

### Immunohistochemical evidence of complex breakdown of axonal cytoskeleton and transport processes

**Figure 2:**
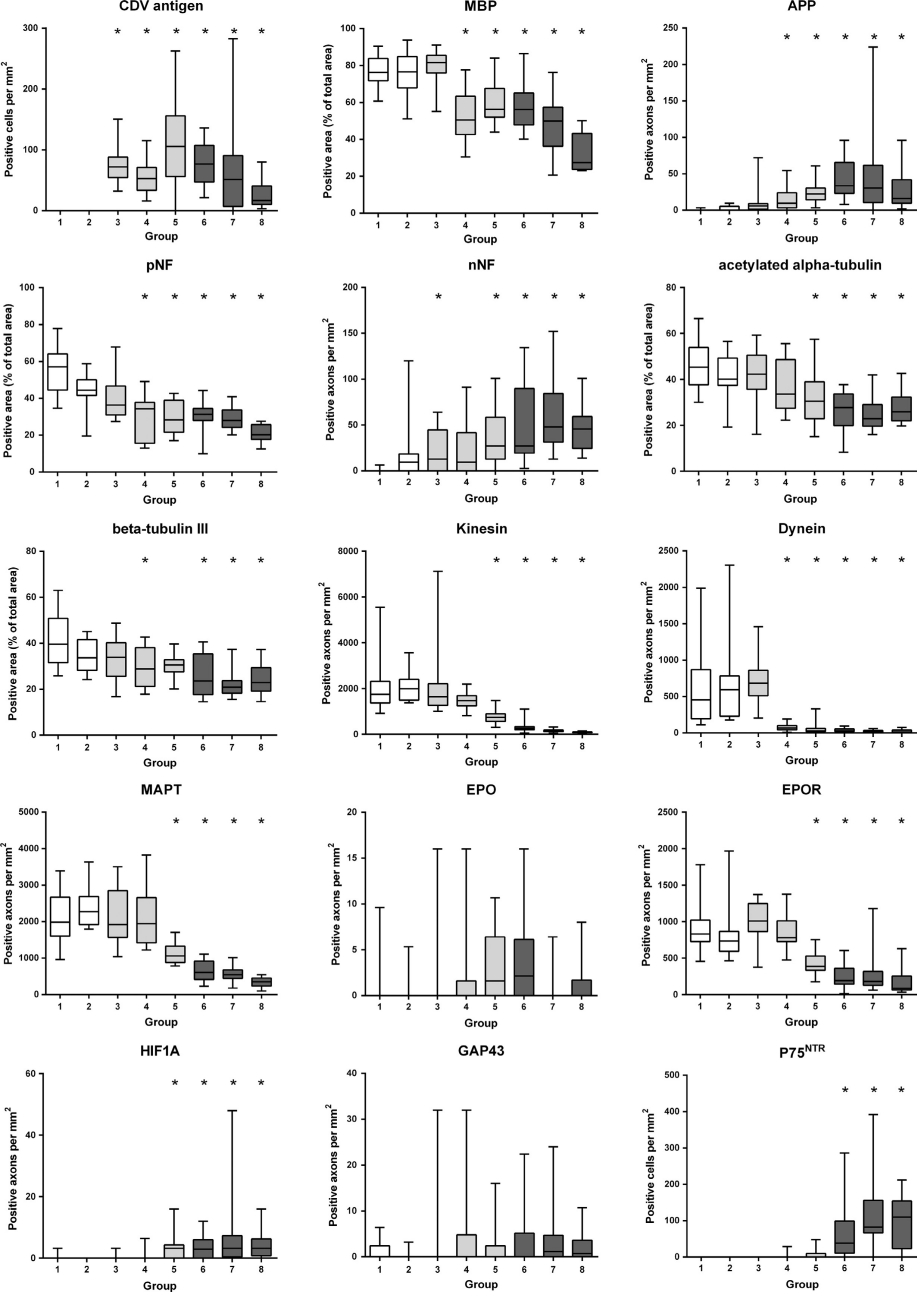
Boxplots illustrating the results of the quantitative evaluation of immunohistochemical expression of canine distemper virus (CDV) antigen, myelin basic protein (MBP), beta‐amyloid precursor protein (APP), phosphorylated and nonphosphorylated neurofilament (pNF, nNF), acetylated alpha‐tubulin, beta‐tubulin III, kinesin, dynein, microtubule‐associated protein tau (MAPT), erythropoietin, (EPO) and its receptor (EPOR), hypoxia‐inducible factor 1 alpha (HIF1A), growth‐associated protein 43, and p75 neurotrophin receptor (P75^NTR^) during the disease course of distemper leukoencephalitis. Groups: 1‐controls, 2‐normal appearing white matter, 3‐antigen without lesion, 4‐vacuolation, 5‐acute lesions, 6‐subacute lesions without inflammation, 7‐subacute lesions with inflammation, 8‐chronic lesions. Asterisks indicate significant differences (*P* ≤ 0.05) as compared to controls (group 1). *P*‐values of all group‐wise comparisons are given in Table S1.

**Figure 3:**
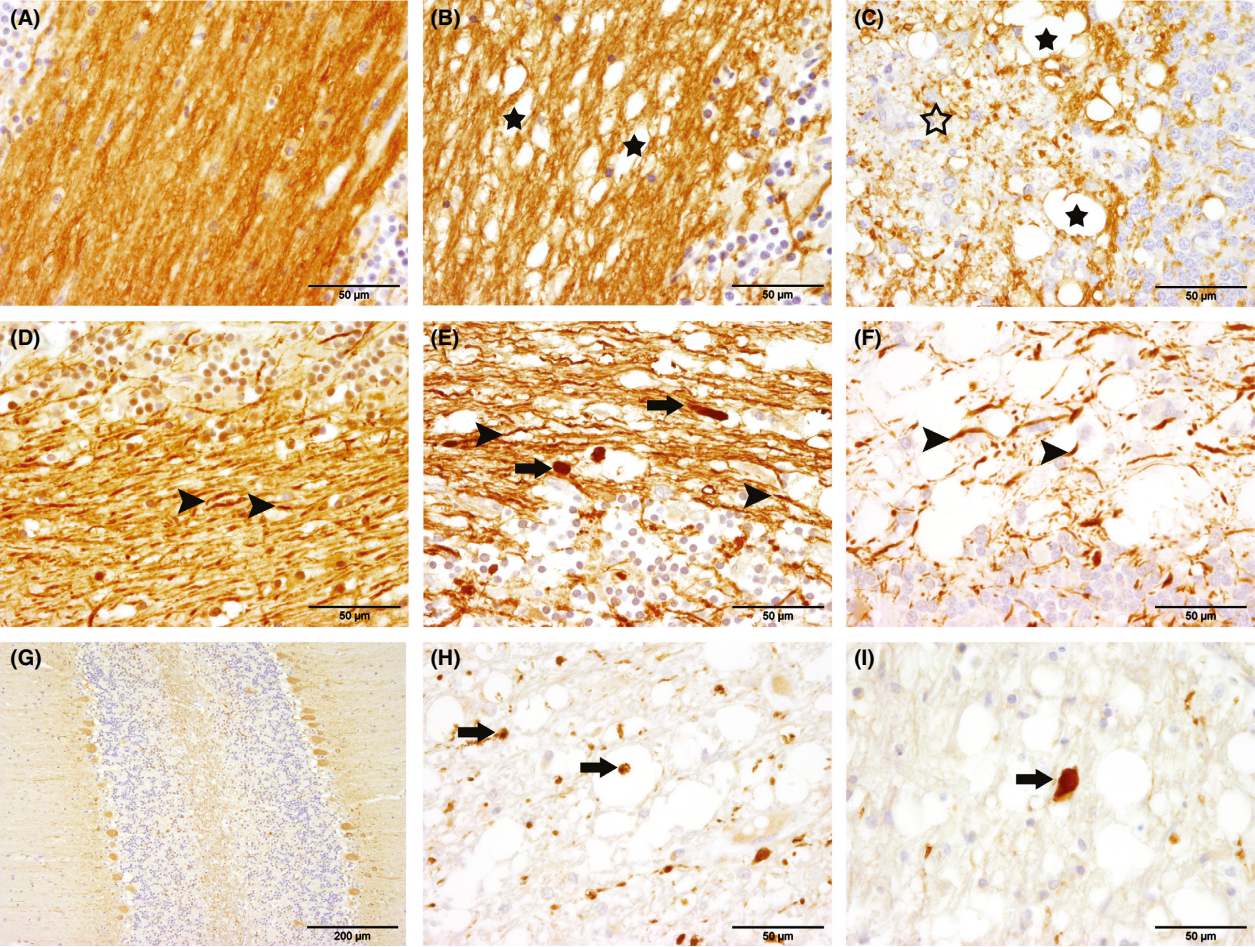
Immunohistochemical detection of myelin basic protein (MBP; A, B, C), phosphorylated (pNF; D, E, F) and nonphosphorylated neurofilament (nNF; G, H, I) in the cerebellum of dogs with canine distemper demyelinating leukoencephalitis (CDV‐DL). The left column corresponds to controls, while the middle column shows early (acute) lesions and the right column displays advanced (subacute to chronic lesions). (A) cerebellar arborization of a control animal (group 1) with dense myelin in the white matter. (B) cerebellar arborization of an acute CDV‐DL lesion (group 5) showing decreased density of myelin with formation of vacuoles (black asterisks). (C) cerebellar arborization of a chronic lesion (group 8) characterized by vacuoles (black asterisks) and marked myelin loss (open asterisk) with inflammatory infiltrates in the white matter. (D) white matter of a control animal (group 1) with densely packed pNF‐positive axons (arrows) partly projecting into the granular layer. (E) acute CDV‐DL lesion (group 5) showing loosening of pNF‐positive axons (arrowheads) and accumulation of pNF in swollen axons (arrows). (F) subacute CDV‐DL lesion (group 7) with extensive loss of pNF‐positive axons (arrows). (G) Cerebellar arborization of a control animal. The cytoplasm of the Purkinje cells stains weakly positive for nNF, while no positive axons are evident in the white matter. (H) acute CDV‐DL lesion (group 5) in the cerebellar white matter with numerous nNF‐positive axons, indicating axoskeletal defects (arrows). (I) subacute CDV‐DL lesion (group 7) with an intralesional, swollen nNF‐positive axon (arrow). Immunohistochemistry. Avidin‐biotin‐peroxidase complex method with 3,3′‐diaminobenzidine as chromogen.

**Figure 4:**
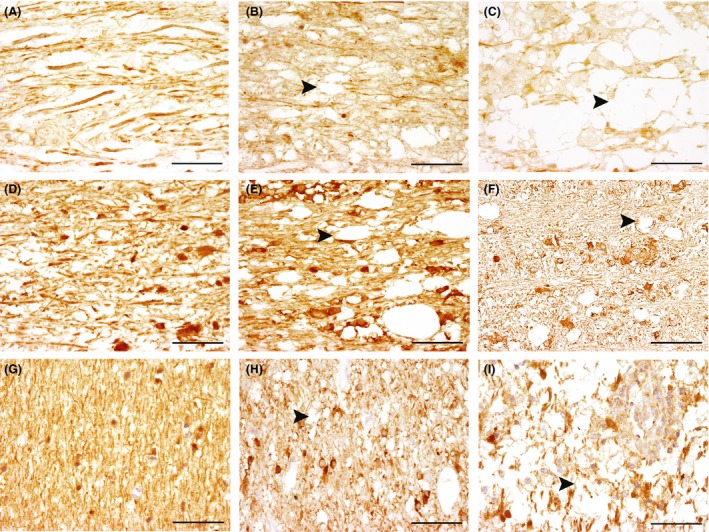
Expression of dynein (A–C), kinesin (KIF5A; D–F), and microtubule‐associated protein tau (MAPT; G–I) the cerebellar white matter of controls and dogs with canine distemper demyelinating leukoencephalitis (CDV‐DL). (A) control animal (group 1) with evidence of strongly dynein‐positive axons. (B) acute CDV‐DL lesion (group 5) with decreasing dynein expression in the axons (arrowhead). (C) subacute CDV‐DL lesion (group 7) with evidence of intralesional gitter cells and extensive reduction in dynein‐positive axons (arrowhead). (D) control animal (group 1) showing densely packed KIF5A‐positive axons in the white matter and a positive cytoplasmic signal in some glial cells. (E) acute CDV‐DL lesion (group 5) with formation of vacuoles and decreased amounts of KIF5A‐positive axons (arrowhead) and a positive cytoplasmic signal in some glial cells. (F) chronic CDV‐DL lesion (group 8) showing severe reduction in KIF5A‐positive axons (arrowhead) and a positive cytoplasmic signal in some glial cells. (G) control animal (group 1) with densely arranged MAPT‐positive axons. (H) acute CDV‐DL lesion (group 5) showing a positive cytoplasmic signal in some glial cells and reduced amounts of MAPT‐positive axons (arrowhead). (I) chronic lesion (group 8) showing inflammatory infiltrates with a positive cytoplasmic signal in some glial cells and severe reduction of MAPT‐positive axons (arrowhead). Immunohistochemistry. Avidin‐biotin‐peroxidase complex method with 3,3′‐diaminobenzidine as chromogen. Scale bars: 50 *μ*m.

Figure 2 summarizes the results of the statistical analysis of the investigated antigens. CDV antigen was detected in all lesions (group 3–8), but not in controls (group 1) and lesions without antigen (group 2), as expected. A decrease in MBP‐positive area was notable (*P* < 0.001), beginning in group 4, and progressing toward the advanced disease phase in groups 6–8 (Figs. 2, 3). Significant axonal accumulation of APP (*P* < 0.001) was evident, beginning in group 4. Moreover, significantly increased numbers of APP‐positive axons were noted in advanced lesions of group 7 compared to earlier lesions (group 2 and 3), indicating progressive disturbances in axonal transport mechanisms. Lesions affecting the axonal cytoskeleton were characterized by a highly significant decrease in the overall axonal density as detected by persistently diminished pNF‐positive area (*P* < 0.001), beginning in group 4 and ongoing to group 8 (Figs. 2, 3). While axons of control dogs generally lacked expression of nNF, there were increasing numbers of nNF‐positive axons (*P* < 0.001), beginning as early as in group 3 as compared to controls (Figs. 2, 3). Both investigated tubulins, acetylated alpha‐tubulin and beta‐tubulin III, displayed a highly significant loss of expression in advanced lesions of groups 5–8 (*P* < 0.001). While in controls, the vast majority of axons displayed a strong positive signal for KIF5A, dynein, and MAPT, a decreased expression of these motor proteins and the microtubule‐associated protein was evident (all antigens: *P* < 0.001), beginning in group 4 (dynein and MAPT) and group 5 (KIF5A), respectively, as compared to controls and the earlier lesions (Figs. 2, 4).

### Limited expression of proteins involved in axonal regeneration and Schwann cell remyelination during CDV‐DL

**Figure 5:**
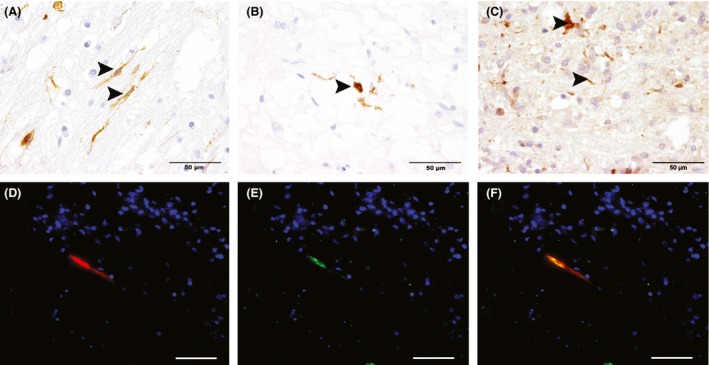
Immunohistochemical demonstration of expression of p75 neurotrophin receptor (p75^NTR^, A), periaxin (PRX) (B), and hypoxia‐inducible factor 1 alpha (HIF1A; C) in canine distemper leukoencephalitis (CDV‐DL) lesions in the cerebellar white matter. (A) Few intralesional bipolar cells show immunoreactivity for p75^NTR^ (arrowheads), indicative of premyelinating Schwann cells, in a chronic lesion of CDV‐DL. (B) Single PRX‐positive structures (arrowheads) in close proximity to dilated myelin sheaths in a chronic lesion of CDV‐DL, indicating Schwann cell‐mediated remyelination. (C) A chronic lesion of CDV‐DL with inflammatory infiltrates and few intralesional axons, expressing HIF1A (arrowheads). Immunohistochemistry. Avidin‐biotin‐peroxidase complex method with 3,3′‐diaminobenzidine as chromogen. (D–F) Double immunofluorescence staining for P0 (red, A) and PRX (green, B) in a chronic lesion of canine distemper reveals that PRX‐positive Schwann cells present within the lesion coexpress myelin protein 0 (C). Nuclear counterstaining (blue) with bisbenzimide. Scale bars: 50 *μ*m.

Axonal expression of HIF1A expression was scant in controls, but the number of positive, predominantly swollen axons increased significantly in advanced lesions (groups 5–8) as compared to controls and earlier lesions (*P* < 0.001; Figs. 2, 5). In contrast, the number of axons expressing EPO was generally low in all groups including advanced CDV lesions. Even though the global Kruskal–Wallis test indicated significant differences between the groups (*P* = 0.04), none of the pair‐wise comparisons reached the level of significance. In contrast, EPOR expression was strongly present in a large number of axons in controls. However, the number of EPOR‐positive axons revealed a continuous decrease (*P* < 0.001), which was particularly evident in groups 5–8 compared to controls. Axonal expression of GAP43, indicative of axonal regeneration, failed to reach the level of significance in dogs with CDV‐DL (*P* = 0.104). Single positive axons were observed in all groups including the controls. There was significant spontaneous occurrence of bi‐ to multipolar cells, expressing p75^NTR^ in subacute and chronic CDV‐DL lesions, suggestive of premyelinating Schwann cells. No or only very few p75^NTR^‐expressing cells were seen in group 1–5, whereas there was a significant increase in the number of p75^NTR^‐positive cells in groups 6–8 (Figs. 2, 5).

The adjusted *P*‐values for each pair‐wise comparison between the different lesion groups are given within Table S1.

Double immunofluorescence was performed on selected sections in order to characterize the phenotype of these cells in more detail (Kegler et al. 2015). None of the p75^NTR^‐positive cells coexpressed SOX2, GFAP, PDGFR‐alpha, and GAP43, respectively (Fig. S1). In order to identify Schwann cell remyelination, immunohistochemistry for PRX, a myelin protein restricted to peripheral myelin (Scherer et al. 1995; Mirsky et al. 2008), was performed. In only two lesions 121 (1.65%), one of group 6 and one of group 8, there was evidence of PRX‐positive, round to oval structures, suggestive of myelinating Schwann cells (Fig. 5). Representative immunofluorescence staining of these two lesions revealed that these PRX‐positive cells were additionally double positive for myelin protein 0 (Fig. 5).

### Strong interdependence of breakdown in anterograde and retrograde transport systems

Calculation of the Spearman's rank correlation coefficient was performed in order to reveal potential codependencies of the antigens investigated by immunohistochemistry. Only correlation coefficients of *r* > 0.7 or *r* < −0.7 and *P* ≤ 0.05 were considered biologically relevant. There was strong positive correlation of axonal dynein expression with expression of KIF5A (*r* = 0.752; *P* < 0.001) and MAPT (*r* = 0.745; *P* < 0.001). KIF5A expression was similarly strongly positively correlated with the expression of MAPT (*r* = 0.843; *P* < 0.001). Moreover, there was strong positive correlation of EPOR expression with both KIF5A (*r* = 0.767; *P* < 0.001) and MAPT (*r* = 0.732; *P* < 0.001). In addition, the number of p75^NTR^ positive bi‐ to multipolar cells negatively correlated with axonal expression of KIF5A (*r* = −0.753; *P* < 0.001) and MAPT (*r* = −0.771; *P* < 0.001).

### Transcriptome data reflect complex axonal pathology but indicate delayed counterregulation of several axonal genes in chronic CDV‐DL lesions

In order to reveal, whether the morphological changes could also be recapitulated on the transcriptome level, we analyzed the expression of a manually generated literature‐based list of genes (Table S2), involved in axonal processes, derived from a publically available microarray data set of brain tissue from an independent CDV‐DL study (Ulrich et al. 2014). Out of the total number of 57 genes (135 probe sets; Table S2), 30 unique canine gene symbols represented by 50 probe sets were identified as being differentially regulated (*P* < 0.05) in at least one pair‐wise comparison (Table 2). There were only slight changes in acute lesions as compared to controls. Seven probe sets were slightly down‐regulated as compared to controls. However, a mild up‐regulation of multiple probe sets encoding for subunits of protein phosphatases (*protein phosphatase 2, catalytic subunit, alpha isozyme; protein phosphatase 2, regulatory subunit A, alpha; protein phosphatase 2, regulatory subunit A, beta*) was evident in acute lesions as compared to controls (Table 2). The most prominent transcriptional changes were evident in the pair‐wise contrast of subacute lesions compared to controls. Here, a total number of 37 out of the 50 probe sets were differentially expressed and all but one differentially expressed probe set, annotated by *dynein, axonemal, heavy chain 10*, exhibited a down‐regulation (Table 2). The probe sets encoded for multiple axonal genes including *APP*, dynein components such as *DNAH1*, kinesins, microtubule components, neurofilament subunits, tubulins, and subunits of *protein phosphatase 2A* with fold changes ranging from −3.78 (*NF, medium polypeptide*) to −1.14 (*kinesin light chain 2*).

Interestingly, the pair‐wise comparison of chronic lesions compared to subacute lesions resulted in a total number of nine differentially expressed probe sets, representing seven unique genes (Table 2). Out of these, only two genes encoding for tubulins exhibited a down‐regulation while the remaining five genes (*kinesin heavy chain member 2A; dynein light chain 1, cytoplasmic‐like; NF, heavy polypeptide; NF, medium polypeptide; protein phosphatase 2, regulatory subunit B, beta*) were up‐regulated. *Neurofilament, medium polypeptide* (*NEFM*) was the gene with the highest fold change (3.13) in comparison to subacute lesions. No genes encoding for factors involved in axonal regeneration such as *HIF1A*,* EPO*,* EPOR*, and *GAP43*, were differentially expressed on the transcriptome level.

## Discussion

**Figure 6:**
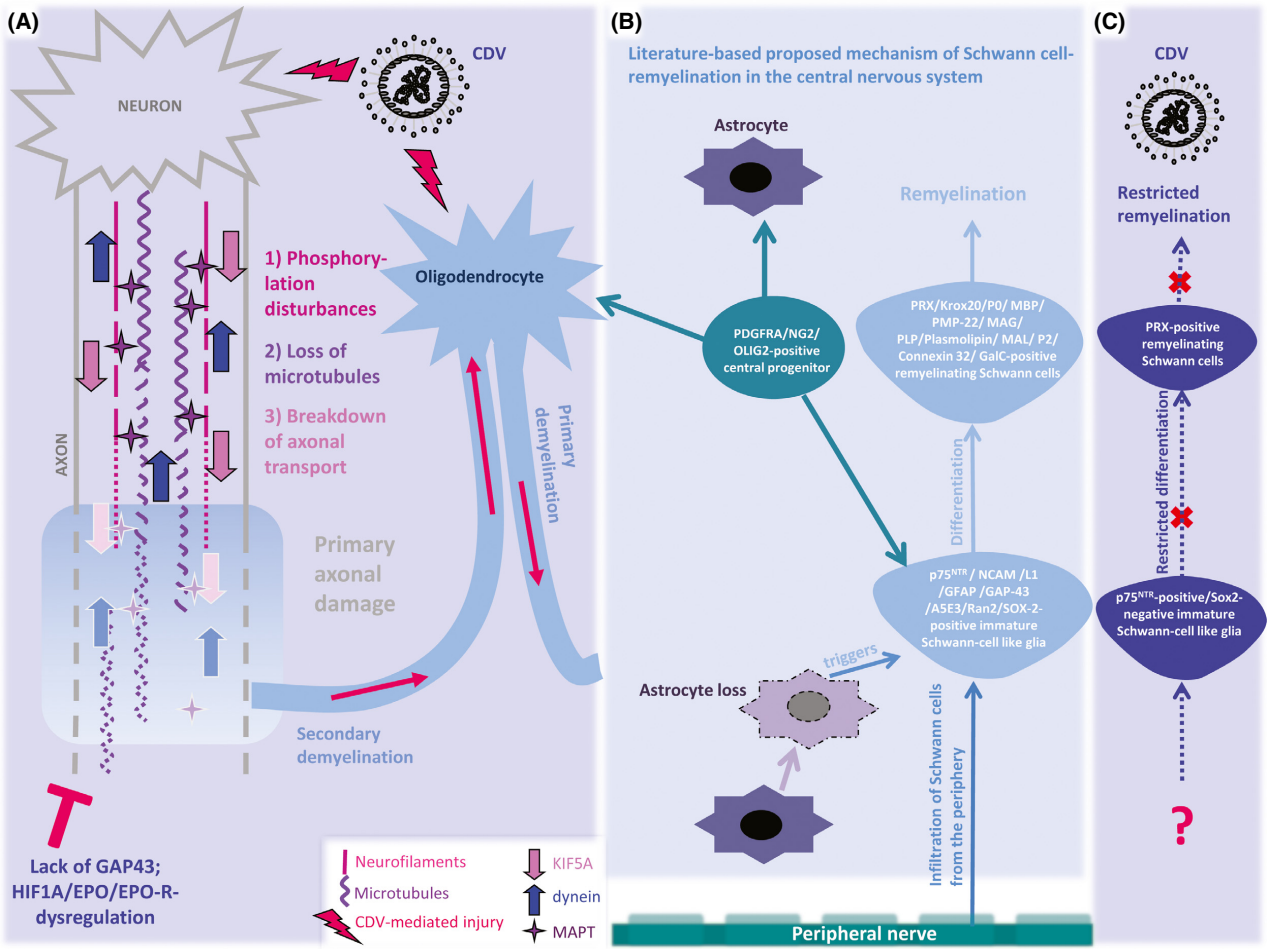
Schematic display of the proposed pathogenesis of primary versus secondary demyelination, axonal damage, and Schwann cell remyelination in canine distemper virus leukoencephalitis (CDV‐DL). (A) Primary axonal damage in CDV‐DL is characterized by severe disturbances in the neurofilament compartment with phosphorylation disturbances, followed by loss of microtubules and decreased axonal transport capability as demonstrated by reduced expression of microtubule‐associated protein tau (MAPT), kinesin (KIF5A), and dynein. Demyelination in part arises secondarily and results in myelin loss (secondary demyelination). In parallel, CDV‐mediated injury to oligodendrocytes and the myelin sheaths may lead to primary demyelination of uninjured axons. Despite detection of few hypoxia‐inducible factor 1 alpha (HIF1A)‐positive axons, attempts of axonal regeneration as determined by lack of growth‐associated protein‐43 (GAP43)‐positive axons remain inefficient, presumably due to dysregulation of regenerative factors such as erythropoietin (EPO) and its receptor (EPO‐R). (B) According to published observations in experimental rodent models for demyelinating diseases (Zawadzka et al. 2006), basic differentiation principles of the Schwann cell lineage in the peripheral nervous system (Mirsky et al. 2008), and recent observations in canine idiopathic granulomatous meningoencephalitis (GME; Kegler et al. 2015), the proposed mechanisms of Schwann cell‐mediated remyelination include (1) infiltration of Schwann cells from the peripheral nerves and/or meninges. In addition, (2) PDGFRA/NG2/OLIG2‐positive central precursor cells, possibly identical to oligodendrocyte precursor cells, have been shown to differentiate into oligodendrocytes and astrocytes, but additionally represent an endogenous central source for Schwann cells (Zawadzka et al. 2006). Schwann cells have been demonstrated to occur mainly in areas devoid of astrocytes. Immature Schwann cells are positive for a variety of markers including p75^NTR^ and SOX2, as shown for instance in GME (Kegler et al. 2015). They differentiate into myelinating Schwann cells, which among other markers express periaxin (PRX) and myelin protein zero (P0). (C) In CDV‐DL, despite occurrence of a relatively high number of p75^NTR^, but SOX2‐negative immature Schwann cell‐like glia, differentiation of these cells into fully competent remyelinating periaxin (PRX)‐positive Schwann cells is restricted to few individual lesions. The causes for these discrepancies between CDV‐DL and other animal models and diseases, respectively (B), in terms of insufficient Schwann cell remyelination in CDV‐DL remain to be investigated. Moreover, even though axonal damage has been associated with the occurrence of p75^NTR^‐positive glia in CDV‐DL, additional factors triggering the occurrence of p75^NTR^‐positive glia in CDV‐DL still need to be identified. Moreover, the clinical relevance of Schwann cell‐mediated remyelination in CDV‐DL needs to be clarified in future studies.

Though degenerative processes of both axons and myelin during CDV‐DL have been detailed in various studies, there is a considerable knowledge gap on the underlying molecular pathomechanisms. This study highlights for the first time, the complex sequence of axonal degeneration in CDV‐DL and provides hints for sparse endogenous regenerative processes in the late disease phase. A schematic figure summarizes the proposed pathogenesis of CDV‐DL with special emphasis upon axonal pathology and Schwann cells and their impact in de‐ and remyelination, respectively (Fig. 6).

In this study, we chose a multidirectional approach using both immunohistochemistry and analysis of gene expression. A drawback of this approach is represented by the fact that the immunohistochemical and the microarray data are based on differing group assignments and were performed on different populations of study subjects, thus preventing direct comparisons and correlation analyses of genes and proteins. However, the fact that the immunohistochemical findings were vastly mirrored by the transcriptional data demonstrates the usefulness of this combined approach and basically confirms previous findings by using different populations and methods. Moreover, this approach allowed an investigation of a broader population of individuals.

On the protein level, this study substantiated previous observations of a relatively early onset of axonal damage in CDV‐DL substantiating the current hypothesis of primary axonal damage with secondary demyelination in this disease (Seehusen and Baumgärtner 2010). In TMEV‐IDD, early breakdown of multiple transport systems is believed to initiate NF accumulation, and local NF dephosphorylation rather seems to play a subordinate role (Kreutzer et al. 2012). Even though axonal expression of the investigated tubulins as well as MAPT similarly decreased in CDV‐DL lesions, axonal expression of nNF interestingly preceded these changes in this study. Certain phosphatases such as protein phosphatase 2ac (PP2AC) and PP2AA mediate local phosphorylation and dephosphorylation events of NF, respectively (Veeranna et al. 1995; Kreutzer et al. 2012). Interestingly, this study revealed a mild up‐regulation of multiple genes encoding for subunits of this protein phosphatase in acute lesions as compared to controls (Table 2). In contrast, transcription of protein phosphatase 2 complex members is mildly down‐regulated in TMEV‐IDD (Kreutzer et al. 2012). Thus, the complementary expression of both phosphoforms of neurofilaments in CDV‐DL might in fact, in part, be explained by early and transient local disturbances in phosphorylation processes. Based on these immunohistochemical observations in this study, NF phosphorylation disturbances seem to represent an initial event in CDV‐DL, which is subsequently followed by a loss of microtubules and transport disturbances, indicating that the latter rather represents a consequence than the cause of the cytoskeletal defects. Immunohistochemistry demonstrated reduced axonal expression of KIF5A and dynein in axons. The pivotal role of certain kinesin superfamily proteins was similarly implicated in MS pathology, as demonstrated by significant reduction in *KIF5A*,* KIF21B,* and *KIF1B* mRNA expression and KIF5A protein expression in gray matter (Hares et al. 2013). Similarly, both kinesin and dynein components are down‐regulated in TMEV‐IDD (Kreutzer et al. 2012). Moreover, in this study, the expression of both motor proteins exhibited a strong correlation with each other and the expression of MAPT, indicating a strong interdependence of breakdown of anterograde and retrograde transport systems. Similarly, the present data demonstrate the decreasing expression of alpha‐ and beta‐tubulins in parallel with reduced expression of MAPT, which is crucial for the interaction of microtubules, motor proteins, and NF (Weingarten et al. 1975; Gotow 2000; Shah et al. 2000). The parallel reduction in the expression of the aforementioned cytoskeletal and transport components indicates that axonal transport is disturbed at multiple levels in this entity. It cannot be completely ruled out that the down‐regulated expression of the investigated axonal proteins is in part the consequence of axonal loss. However, the asynchronous reduction in the investigated markers (Fig. 2), especially NF, as well as the lack of strong correlation between some of the antigens implicates that the observed breakdown of cytoskeletal and transport‐related proteins cannot solely be explained by loss of axons.

Multiple axonal genes including genes encoding for dynein components, kinesins, microtubule components, NF subunits, and tubulins were down‐regulated (Table 2), thus vastly reflecting and substantiating the immunohistochemical findings. However, an unexpected finding on the transcriptome level was the fact that the pair‐wise comparison of chronic, inflammatory lesions compared to subacute, demyelinating lesions demonstrated a significant up‐regulation of five genes (*kinesin heavy chain member 2A; dynein light chain 1, cytoplasmic‐like; NF, heavy polypeptide; NF, medium polypeptide; protein phosphatase 2, regulatory subunit B, beta*) in chronic lesions compared to subacute lesions. This finding might indicate a counterregulatory or adaptive mechanism in terms of potential regenerative events. In fact, axonal regeneration is dependent on an accumulation of disorganized NFs and MTs, resembling a growth cone (Gotow 2000; Sunil et al. 2012; Shea and Lee 2013; Liu and Dwyer 2014). Similarly, axonal motor protein accumulation represents an effort to clear the site of injury from cytoskeletal constituents and thus allows an aligned regrowth (Motil et al. 2006). However, even though the transcriptome data indicated a counterregulation of the genes encoding for these proteins in advanced lesions, immunohistochemistry failed to detect any enhanced expression of KIF5A, dynein, or NF in chronic lesions. This discrepancy could either be explained by insufficient translation or by protein amounts below the detection level in the investigated lesions. Enhanced expression of certain endogenous factors such as EPO, EPOR, and HIF1A is considered to contribute to neuroprotection and neuroregeneration, respectively (Bergeron et al. 1999; Ehrenreich et al. 2007; Toth et al. 2008; Hagemeyer et al. 2012). While there was no change in these genes on the transcriptome level, this study demonstrated axonal up‐regulation of HIF1A in axons of CDV‐DL lesions (groups 5–8) by immunohistochemistry. The transcriptional activator HIF1A is crucially involved in the regulation of EPO transcription (Shein et al. 2005; Baltaziak et al. 2013). Thus, we hypothesized that the observed axonal up‐regulation of HIF1A on the protein level might have led to simultaneous up‐regulation of EPO and its receptor. Interestingly, the number of EPO‐positive axons was similar when applying respective group‐wise comparisons. Axonal EPOR expression even revealed a down‐regulation (decreasing amounts of EPOR‐positive axons during disease progression).

Similarly, axonal expression of GAP43, a widely used marker for axonal regeneration, failed to reach the level of significance in CDV‐DL, indicating that the demonstrated cytoskeleton breakdown might have led to failing transport of GAP43 to the injury site (Bisby 1988). This is in contrast to other CNS diseases including trauma and MS, which are characterized by axonal immunoreactivity for GAP43 (Li et al. 1996; Bock et al. 2013; Schirmer et al. 2013). Interestingly, in MS, the number of GAP43‐positive axons within demyelinated plaques correlates with the number of macrophages (Schirmer et al. 2013). Also in dogs with SCI, there is significant axonal expression of GAP43 in parallel with an immune response, dominated by macrophages (Spitzbarth et al. 2011; Bock et al. 2013). As macrophages similarly play a pivotal role in CDV‐DL (Stein et al. 2008), the discrepancies in terms of lacking up‐regulation of GAP43 in CDV‐DL remain undetermined. Speculatively, there might be differences in the polarization of macrophages in CDV‐DL compared to the aforementioned diseases, consequently favoring or suppressing axonal regeneration. In fact, the polarization of macrophages into either the M1 or M2 phenotype has been demonstrated to critically influence the capacity of axonal regeneration (Gensel et al. 2009; Mikita et al. 2011). Whether such polarization differences in macrophages plays a role in CDV‐DL remains to be investigated in further studies.

Remyelination has been demonstrated in various demyelinating diseases. However, in some diseases, efficient oligodendrocytic remyelination is believed to fail due to insufficient differentiation of precursors, as for instance shown in TMEV‐IDD (Ulrich et al. 2008; Sun et al. 2015). Besides oligodendrocytic remyelination, demyelinated CNS axons can be remyelinated by Schwann cells under certain circumstances (Fig. 6), even though the functional consequence of Schwann cell‐mediated remyelination in vivo is not understood in detail (Blakemore 2005; Crawford et al. 2013). However, suggesting a restoring function, Schwann cell‐ mediated remyelination of demyelinated axons in ethidium bromide‐induced spinal cord demyelination in rats has shown to be associated by restoration of successful conduction in most axons (Felts and Smith 1992). Peripheral‐type myelination of demyelinated CNS axons by Schwann cells has been reported in various CNS diseases (Ghatak et al. 1973; Itoyama et al. 1983; Ulrich et al. 2008; Powers et al. 2013; Kegler et al. 2015), but has so far not been demonstrated in CDV‐DL. P75^NTR^ expression by glial cells has been described in dogs suffering from naturally occurring CDV‐DL, highlighting this molecule as a potential candidate for endogenous regenerative events following CDV infection (Imbschweiler et al. 2012). In this study, the highest number of p75^NTR^‐positive glia was seen in dogs with subacute lesions with remarkable inflammation, thus substantiating previous observations (Imbschweiler et al. 2012). Early axonal damage has been proposed as an initial mechanism that triggers the occurrence of these cells, whose exact origin and nature remain unknown (Fig. 6; Imbschweiler et al. 2012). In the CNS, Schwann cells are mainly observed in areas devoid of astrocytes (Fig. 6; Itoyama et al. 1983; Blakemore 2005; Zawadzka et al. 2010); however, the source of Schwann cells in the CNS is still a matter of debate. While peripheral nerves and meninges have long been regarded as the major source of these cells, it has similarly been demonstrated that PDGFRA/NG2‐positive OPCs (oligodendrocyte precursor cells), besides oligodendrocytes and astrocytes, are capable of giving rise to Schwann cells (Fig. 6; Zawadzka et al. 2010).

Glial immunoreactivity for p75^NTR^ during CDV‐DL indicates a Schwann cell‐like phenotype of these cells and previous reports have collectively referred these cells to as potentially regeneration‐promoting aldynoglia (Gudino‐Cabrera and Nieto‐Sampedro 2000; Orlando et al. 2008; Imbschweiler et al. 2012). Substantiating previous observations, the number of p75^NTR^‐positive cells negatively correlated with axonal KIF5A and MAPT expression, indicating a strong interdependence of axonopathy and occurrence of these cells. However, manifest Schwann cell remyelination has not been reported in CDV‐DL so far. Thus, we investigated the expression of PRX, a protein, which is, similar to myelin protein 0, restricted to peripheral myelin (Scherer et al. 1995; Mirsky et al. 2008). In fact, though only restricted to two lesions (1.65% of all lesions), immunohistochemistry demonstrated expression of PRX and thus for the first time very limited Schwann cell‐mediated remyelination in CDV‐DL lesions. Moreover, these cells were double positive for myelin protein 0, thus substantiating formation of peripheral myelin in these lesions.

In a recent study upon canine GME (granulomatous meningoencephalitis), an idiopathic inflammatory condition, characterized by intense perivascular lymphohistiocytic inflammation p75^NTR^‐positive cells, morphologically resembling the cells, which were observed in this study, coexpressed the transcription factor SOX2 (Kegler et al. 2015) As in peripheral degenerative neuropathy, p75^NTR^‐expressing cells similarly colocalize with SOX2 in GME. Thus, it was suggested that the cells observed in GME might represent dedifferentiated Schwann cells (Kegler et al. 2015). In parallel, GME is characterized by robust Schwann cell remyelination, as demonstrated by numerous PRX‐ and P0‐positive mature myelinating Schwann cells, which are strikingly associated with the presence of p75^NTR^/SOX2‐expressing Schwann cells (Kegler et al. 2015). Interestingly and contrary to the findings in GME, we did not observe any colocalization of p75^NTR^ and SOX2 in this study, implying that the detected p75^NTR^‐positive cells in CDV‐DL might exhibit a different phenotype compared to the cells in GME which might explain the comparatively limited Schwann cell remyelination in CDV‐DL. Substantiating this finding, there is no evidence of transcriptional regulation of genes encoding for peripheral‐type myelin proteins in CDV‐DL lesions (Ulrich et al. 2014). Dissecting the molecular differences between these disease entities will thus represent an interesting topic for future studies, which might help to elucidate, whether the differentiation of Schwann cells into myelinating cells during CDV‐DL is potentially blocked by a differing microenvironment. In summary, the present results demonstrated that early disturbances of cytoskeletal constituents such as NF may subsequently lead to a complex breakdown of axonal transport systems, as demonstrated by reduced expression of motor proteins, MAPT, and tubulins. The relative lack of axonal regeneration on the protein level might be explained by an insufficient transport of neurotrophic factors and an adverse dysregulation of potentially regeneration‐enhancing factors. However, it remains to be determined, whether the detected transcriptional up‐regulation of genes encoding for cytoskeletal and motor protein components in the late phase might result in effective axonal regrowth in more advanced lesions or a more beneficial microenvironment. Overall, these findings may have important implications for future therapeutic approaches, designed to enhance the limited intrinsic regenerative capacity during demyelinating diseases.

## Conflict of Interest

The authors declare no conflicts of interest.

## Supporting information


**Figure S1.** Double immunofluorescence staining in a chronic lesion of a representative case of canine distemper. P75^NTR^ (red) is not coexpressed with Sox‐2 (**A‐C**). Note nuclear signal of Sox2 (green, arrow; **B, C**). There is no colocalization of p75^NTR^ with glial fibrillary acidic protein (GFAP, green; **D‐F**), platelet‐derived growth factor receptor (PDGFR)‐*α* (green, arrow; **G‐I**), and GAP43 (**J‐L**). Nuclear counterstaining (blue) with bisbenzimide. Scale bars: 20 *μ*m (A‐F; J‐L); 100 *μ*m (G‐I).

**Table S1.** The excel table gives the adjusted *P*‐values for each pair‐wise comparison of the immunohistochemical data. The values were retrieved by global Kruskal–Wallis test, followed by post hoc group‐wise comparisons with alpha adjustment for multiple testing.

**Table S2.** The excel table lists the manually generated literature‐based list of 79 canine gene symbols, which were used as input data of genes of interest for axon‐relevant processes (table sheet 1). Table sheet 2 gives the output data of the GC‐RMA normalized log2 transformed expression values of a previously published data set (Ulrich et al. 2014; Accession number: E‐MEXP‐3917), filtered for the genes of interest, as well as the *P*‐values of each pair‐wise Mann–Whitney *U*‐test (group 1 – group 4) for each probe set. Table sheet 3 clarifies the group assignment according to the previously published study (Ulrich et al. 2014).
