# Supplementary figures and images for: Immunohistochemical and transcriptome analyses indicate complex breakdown of axonal transport mechanisms in canine distemper leukoencephalitis
Source: Brain Behav. 2016 May 3;6(7):e00472. doi: 10.1002/brb3.472 (PMC4864272; doi:10.1002/brb3.472)

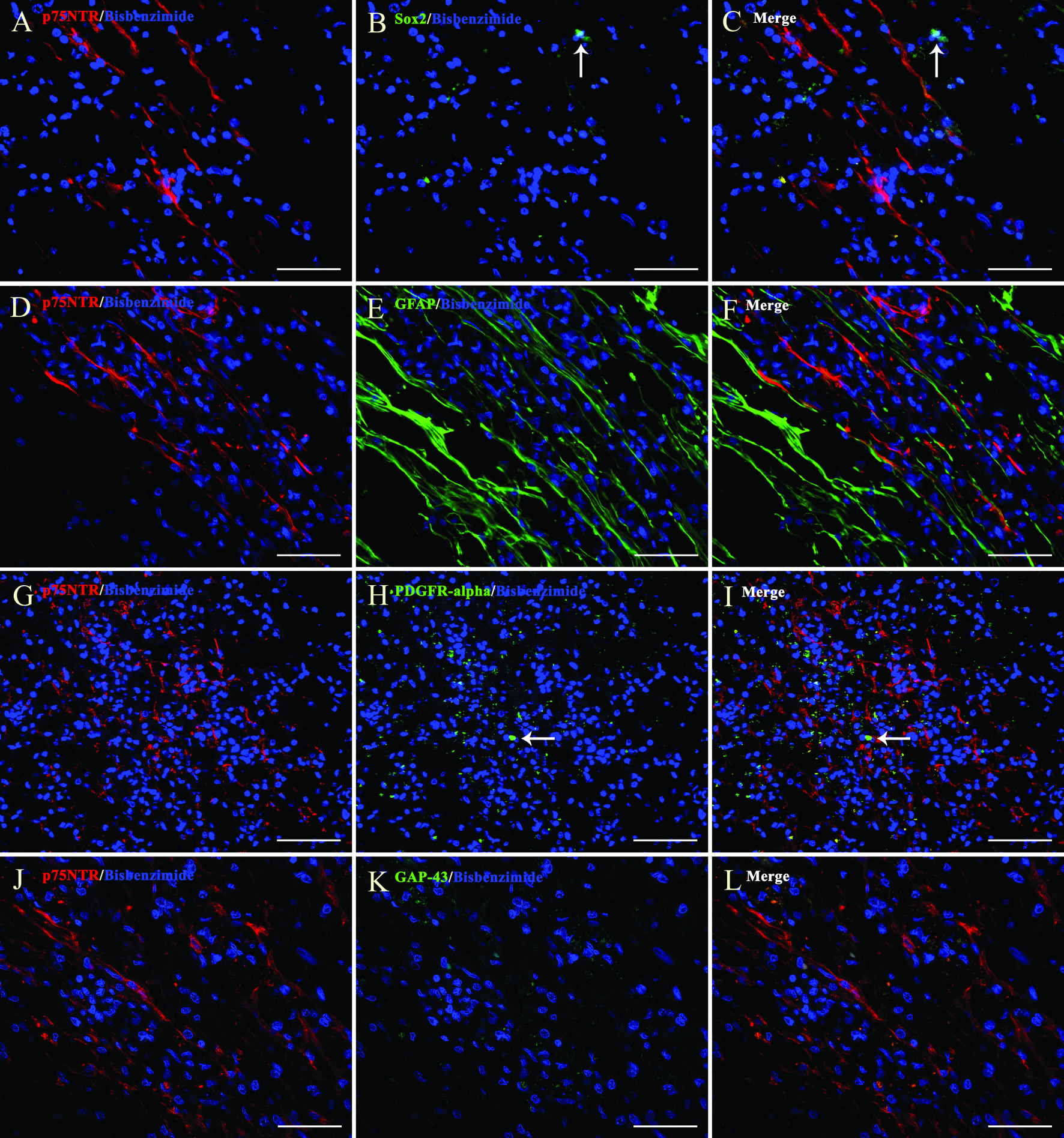

Supplement: Supplementary file 1 — Figure S1. Double immunofluorescence staining in a chronic lesion of a representative case of canine distemper. P75NTR (red) is not coexpressed with Sox‐2 (A‐C). Note nuclear signal of Sox2 (green, arrow; B, C). There is no colocalization of p75NTR with glial fibrillary acidic protein (GFAP, green; D‐F), platelet‐derived growth factor receptor (PDGFR)‐α (green, arrow; G‐I), and GAP43 (J‐L). Nuclear counterstaining (blue) with bisbenzimide. Scale bars: 20 μm (A‐F; J‐L); 100 μm (G‐I). [file BRB3-6-e00472-s001.tif]
